# Three-Dimensional Cell Culture Systems for Studying Hepatitis C Virus

**DOI:** 10.3390/v13020211

**Published:** 2021-01-30

**Authors:** Chui-Wa So, Glenn Randall

**Affiliations:** Department of Microbiology, The University of Chicago, Chicago, IL 60637, USA; cwso@uchicago.edu

**Keywords:** hepatitis C virus, organoids, 3D, polarization

## Abstract

Hepatocytes, the major target of hepatitis C virus (HCV), are highly polarized. HCV infection requires extensive trafficking to distinct subcellular domains in the polarized hepatocyte. Polarized cells and three-dimensional organoids are commonly used to study liver functions and differentiation. Researchers have begun adapting these cell culture models that morphologically and physiologically resemble hepatocytes in vivo to study HCV infection. This review summarizes the use of three-dimensional cell culture systems in studies of HCV infection.

Hepatitis C virus (HCV) is a hepatotropic, enveloped, positive-sense RNA virus of the *Flaviviridae* family. The World Health Organization estimated that 71 million people were infected with HCV in 2015, while HCV-associated hepatocellular carcinoma and cirrhosis accounted for 1 million and 2.5 million deaths, respectively [1]. While the introduction of highly effective direct-acting antivirals has improved HCV therapy, resistance-associated substitutions of NS3, 5A, and 5B have been observed (reviewed in Reference [2]). The need of further research on HCV remains in order to optimize diagnosis, therapy and the development of vaccines [1,3].

Hepatocytes, comprising 60% of the total cells of the liver [4,5], are highly polarized, with two distinct types of membrane domains. The apical domains of adjacent hepatocytes form a continuous bile canaliculus into which bile is secreted, while the basolateral domains are in contact with sinusoids and regulate the exchange of materials with the circulation. Tight junctional proteins play a crucial role in separating the two domains and keeping bile away from the blood circulation. In addition to membrane domains, specific cytoskeletal, endoplasmic reticulum, and Golgi apparatus networks contribute to the complex polarity of hepatocytes [6,7] (reviewed in References [4,8]).

In cell culture-based studies of liver functions, researchers are aware of the importance of hepatocyte polarity. Various human polarized liver cell lines were generated, such as HepG2 and HepaRG (reviewed in Reference [4]). Moreover, human-derived induced pluripotent stem cells [9], human fetal liver cells [10], and bile duct cells isolated from biopsy samples [11] were cultured in extracellular matrices, such as Matrigel and inverted colloidal crystal scaffold, to generate hepatic organoids. The organoids performed liver functions upon transplantation into mice [9]. Lineage and polarity markers, gene expression profiling, and electron microscopy were used to assess differentiation status, polarity, and the degree of similarity between the in vivo systems and the liver [4,12,13,14,15,16,17]. Currently, researchers are exploring the use of the organoids in examining liver toxicity of drugs prior to clinical trials [18,19].

While the use of polarized cells and organoids is not new to the field of liver research, HCV researchers are still exploring ways to generate infection models that morphologically and physiologically resemble the liver. The development of the cell culture system, based on the HCV JFH-1 clone and the human-derived hepatoma Huh-7 cell line, was a breakthrough in HCV research [20,21,22]. Since then, our knowledge of HCV infection in both basic and translational research has greatly advanced. Huh-7 cell line and its derivatives, such as Huh-7.5 and Huh-7.5.1, are widely used in studies of HCV infection. Conventionally, the cells are cultured in two-dimensional (2D) monolayers. The poor polarity of 2D Huh-7 cells has become increasingly appreciated, especially in studies of HCV entry.

HCV entry requires two tight junctional proteins, claudin-1 (CLDN1) and occludin (OCLN) [23,24,25]. In 2D Huh-7 and 7.5 cells, tight junctional markers, ZO-1 and CLDN1, are distributed uniformly on the plasma membrane [13,16,26]. As a result, the cells poorly resemble the bile canaliculus structure, the distinct separation of the apical and basolateral domains, and the retention of bile in the liver. Since CLDN1 and OCLN are known to be essential for HCV entry, they may not exhibit completely conserved functions in nonpolarized cells [27]. Therefore, three-dimensional (3D) cell culture systems that are more physiologically relevant are required to study their roles in HCV entry.

Recently, Huh-7 and 7.5 cells were cultured in Matrigel in HCV studies. Matrigel is a solubilized protein extract from the Engelbreth-Holm-Swarm (EHS) mouse sarcoma [28]. Matrigel was first used to show the requirement of a basement membrane in the differentiation and polarization of human endothelial cells [29]. Molina-Jimenez et al. [16] and Baktash et al. [13] showed polarized localization of apical, basolateral, and tight junctional markers when Huh-7 and 7.5 cells were cultured in Matrigel. Moreover, the bile analog 5-chloromethyfluorescein diacetate (CMFDA) was retained at the apical domains. It suggested that Matrigel-cultured hepatocytes showed functional characteristics of polarization. Using Matrigel-cultured Huh-7.5 cells and single particle fluorescent labeling of HCV (Figure 1), Baktash et al. [13] showed the movement of HCV particles from the basolateral domains to the tight junctions during entry. The data also suggested that HCV particles preferentially internalized at the tight junctions. This entry pattern had not been shown before in 2D Huh-7.5 cells, which do not have distinct apical and basolateral domains. The findings suggest that the use of 3D cell culture systems may reveal unknown mechanisms of HCV infection.

Baktash et al. [13] further showed that epidermal growth factor receptor (EGFR) is dispensable for HCV to migrate to the tight junctions. However, it is required for HCV to recruit clathrin components for endocytosis. Moreover, Brown et al. [30] used the organoid system to characterize the role of two transmembrane proteins, Cd302 and Cr1l, in HCV infection. They are species barriers restricting HCV replication in rodents. Expressing mCd302 or mCd302/mCr1l in Matrigel-cultured Huh-7.5 cells inhibits HCV trafficking to the tight junctions. These studies suggest that Matrigel-based 3D organoid system is useful in understanding HCV infection. Certain steps of HCV entry, such as trafficking to the tight junctions, cannot be evaluated in 2D cells.

HepG2 is another human hepatoma cell line commonly used in HCV studies. Expressing CD81 and miR-122 in HepG2 cells significantly increases their susceptibility to HCV infection [27,31]. HepG2 cells polarize partially. Mee et al. [31] observed that 76% of HepG2-CD81 cells developed apical domains. The apical domains formed bile canaliculus-like structures that retained CMFDA. While CLDN1 was distributed more uniformly on the plasma membrane in nonpolarized cells, it predominantly localized at the apical domains in polarized cells. However, OCLN was only detected at the apical domains in polarized cells. As the cells became more polarized over time, an inverse correlation was observed with respect to the cell culture derived HCV (HCVcc) and HCV pseudoparticle (HCVpp) infection. It suggested a possible influence of hepatocyte polarity on HCV infection, and hence the importance of polarity in HCV studies. Since HepG2 cells do not completely polarize, researchers have attempted to isolate subclones of HepG2 with an enhanced ability to polarize [4,32].

Other than entry, studies using 3D cell culture systems to examine other stages of the HCV life cycle are limited. Benedicto et al. [33] evaluated the roles of clathrin and dynamin in HCV egress and found no significant differences between 2D and Matrigel-cultured Huh-7 cells. Besides the membrane domains, the complex polarity of hepatocytes is displayed in cytoskeletal, endoplasmic reticulum, and Golgi apparatus networks (reviewed in Reference [4]). The effect of cell polarity on other stages of the HCV life cycle, including RNA replication and assembly/egress is yet to be addressed. Liu et al. [34] generated a JFH-1 EGFP reporter virus and validated its infection in Matrigel-cultured Huh-7.5 cells. The reporter virus may be useful in live cell analysis of HCV entry and assembly/egress and antiviral screening in 3D cells.

Mebiolgel is another matrix-based cell culture system that has been used in HCV studies. In contrast to Matrigel, Mebiolgel is a synthetic polymer free of potential biological contaminants. Mebiolgel-cultured Huh-7 cells form spherical clusters and have increased expression of hepatic differentiation markers relative to 2D cells [17]. Using an immortalized hepatocyte cell line HuS-E/2, Aly et al. [35] showed a significant increase in HCV replication when the cells were cultured in Mebiolgel, as compared to 2D cells. The 2D and 3D cells also showed differences in gene expression profiling. The findings highlight the significance of addressing the effect of cell culture systems on HCV infection. Further evaluation of different 3D systems is needed to compare their relevance to infection in vivo.

In addition to extracellular matrices and various cell lines, bioreactor-based approaches have been used to culture hepatocytes in HCV studies. Radial flow and hollow fiber bioreactors are used to mimic the cellular environment in vivo. In the bioreactors, cells attach to semi-permeable capillaries [36,37] or porous glass beads [14]. The cells are nurtured by continuous feeding of fresh medium and removal of toxic metabolites. Aizaki et al. [12] and Kawada et al. [14], respectively, showed that human hepatoma cells FLC-4 and FLC-7 cultured in the radial flow bioreactor were spherical and microvilli-lined. It was in sharp contrast to 2D cells which were flattened and extended with cytoplasmic projections. Using FLC-4 cells cultured in the radial flow bioreactor, Murakami et al. [38] propagated HCV from carriers’ serum samples and showed the change in quasispecies composition. In a study of HCV production, Pihl et al. [39] cultured Huh-7.5 cells in the hollow fiber bioreactor. Since the cells grew at a higher density in the bioreactor than in monolayers [37], higher titers of HCV were produced. When the cells were treated with the NS5A inhibitor daclatasvir, lower infectious viral titers were observed [39]. However, the morphology of Huh-7.5 cells in the hollow fiber bioreactor is yet to be evaluated. Thus far, both the radial flow and hollow fiber bioreactors have been mainly used in the production of HCV stocks. For other viruses, such as human immunodeficiency virus and influenza A virus, the bioreactors have been widely used to determine the pharmacodynamics of antivirals [40,41,42]. The potentials of the systems in studies of HCV life cycle and antivirals are worth exploring.

The use of another bioreactor, the rotating wall vessel, has been explored in HCV studies. While rotating, cells attached to collagen-coated beads experience less shear and turbulence than in flow bioreactors (reviewed in Reference [43]). Sainz et al. [44] showed that Huh-7 cells formed 3D aggregates in the rotating vessel. The cells had higher expression of host factors of HCV entry, including CD81, CLDN1, and OCLN, than 2D cells. How the change in expression affects HCV infection and its relevance to infection in vivo are yet to be addressed.

To conclude, HCV infection, particularly entry, depends on the complex polarity of hepatocytes. While the use of 2D Huh-7 cells have advanced our understanding of HCV infection, the nonpolarized cells may not fully resemble the physiology of hepatocytes in vivo. Development of the optimal 3D systems is obstructed by their relevance and permissiveness to HCV infection. Polarized cell lines, such as HepG2, and matrix or bioreactor-based cell culture are promising 3D infection models. Thus far, they have been used in studies of HCV entry and egress, and showed processes of entry that are not observed in 2D cells. Further studies of the localization and functions of host factors in 3D cells may reveal unknown mechanisms of entry and egress. Besides membrane proteins, other cellular components, such as the endoplasmic reticulum and secretory pathway, also exhibit polarity in hepatocytes. Its effect on HCV replication and assembly is yet to be evaluated.

## Figures and Tables

**Figure 1:**
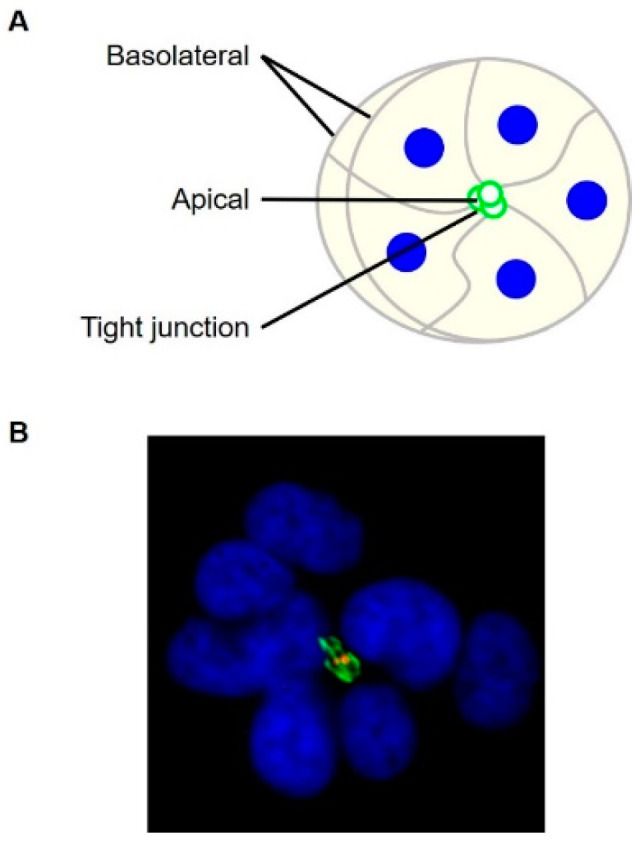
Huh-7.5 cells cultured in Matrigel. (**A**) Diagrammatic of a polarized Huh-7.5 organoid with membrane domains. (**B**) Huh-7.5 cells were grown in Matrigel, infected with DiD-labelled HCV (red) for 90 min, and probed for tight junctional marker ZO-1 (green).

## Data Availability

Not applicable.
